# The Influence of Time of Day on Critical Swimming Speed in Chronotype-Screened (Neither-Type) Well-Trained Male Swimmers

**DOI:** 10.3390/life16091432

**Published:** 2026-08-28

**Authors:** Gökhan Tuna, Cem Kurt, Erkan Günay, Zeynep Bozdoğan Kurt, Raul Ioan Muntean, İsmail Dergaa, Halil İbrahim Ceylan

**Affiliations:** 1 Department of Coaching Education, Kırkpınar Faculty of Sport Sciences, Trakya University, 22030 Edirne, Türkiye; tunagokhan@yandex.com; 2 Department of Kinesiology and Health, College of Health, Education, and Human Services, Wright State University, Dayton, OH 45435-0001, USA; cemkurt35@gmail.com; 3 Department of Coaching Education, Faculty of Sport Sciences, Manisa Celal Bayar University, 45040 Manisa, Türkiye; 4 Department of Sports Health, Faculty of Sport Sciences, Marmara University, 34815 Istanbul, Türkiye; 5 Department of Physical Education and Sport, Faculty of Law and Social Sciences, University “1 Decembrie 1918” of Alba Iulia, 510009 Alba Iulia, Romania; 6 Department of Biological Sciences, High Institute of Sport and Physical Education of Ksar Said, University of Manouba, Manouba 2010, Tunisia; phd.dergaa@gmail.com; 7 Department of Physical Education and Sports Teaching, Faculty of Sport Sciences, Atatürk University, 25240 Erzurum, Türkiye

**Keywords:** critical swimming speed, circadian rhythm, chronotype, diurnal variation, front crawl, time of day

## Abstract

**Background**: Critical swimming speed (CSS) is widely used to prescribe training and monitor endurance-related change, yet it is unclear whether its estimate varies with time of day when chronotype and relevant pre-trial conditions are considered. **Purpose**: To compare CSS at 09:00 h and 17:00 h in well-trained male swimmers with a homogeneous neither/intermediate (N-type) chronotype. **Methods**: Sixteen well-trained male front-crawl swimmers (age 17 ± 1 yr; height 1.78 ± 0.03 m; body mass 69.18 ± 4.56 kg; experience 11 ± 2 yr; weekly swimming volume ≈45 km) completed morning and evening sessions in a randomised, counter-balanced order, separated by 48 h. Each session comprised a 50 m trial followed by a 400 m trial, from which CSS was calculated. Twenty-four-hour energy and carbohydrate intake, forehead skin temperature, resting and post-trial heart rate, the Hooper Index, and Borg CR-10 RPE were also assessed. **Results**: CSS, the pre-specified primary outcome, was significantly higher in the evening than in the morning (morning, 1.46 ± 0.03; evening, 1.47 ± 0.05 m·s^−1^; Δ = +0.68%; *p* = 0.045; d_av = 0.24). The 400 m time was significantly faster in the evening (morning, 265.98 ± 6.25; evening, 264.37 ± 8.07 s; Δ = −0.61%; *p* = 0.047), whereas the 50 m time did not differ (*p* = 0.380). Forehead skin temperature and resting heart rate were higher in the evening (both *p* < 0.001); all other physiological, perceptual, well-being, and dietary comparisons were non-significant. **Conclusions**: A small evening increase in CSS, accompanied by faster 400 m performance, was observed in this homogeneous N-type cohort. The physiological measurements were concurrent observations and do not establish a mechanism. Standardising the time of day of repeated CSS assessment is recommended; larger, sex-balanced, and chronotype-diverse studies are needed before applying a numerical time-of-day correction.

## 1. Introduction

Aerobic endurance is a central determinant of swimming performance, but its expression can vary within and between days. Front-crawl events span approximately 20 s to 60 min and therefore encompass markedly different energetic demands. Within this range, sustainable performance depends on intensities between the anaerobic threshold and maximal aerobic speed [1]. Critical swimming speed (CSS) is a practical estimate of the heavy-to-severe exercise-intensity boundary in swimmers [2,3]. It is used to prescribe training pace, monitor adaptation, and support pacing decisions [4,5]. Consequently, any systematic influence of testing time on CSS could be mistaken for a training-related change when repeated assessments are performed at different times of day.

Athletic performance is shaped by internal factors, including health and training status, and by external factors such as diet, sleep, social context, and the physical environment [6]. Time of day may also influence performance [7,8,9,10] through circadian variation in body temperature, cardiovascular function, substrate metabolism, and hormonal secretion [11,12]. Performance often peaks in the late afternoon, when temperature and several neuromuscular and metabolic processes are elevated, although the magnitude and direction of this pattern vary among athletes and tasks [7,13].

Chronotype describes an individual preference for morning or evening activity [14,15] and is commonly classified as morning-type (M-type), evening-type (E-type), or neither/intermediate-type (N-type) [16]. M-types tend to perform better earlier in the day, E-types later in the day, and N-types show a less extreme temporal preference [17,18]. Several studies have reported morning-to-evening improvements of approximately 1.2–3.5% in 25–200 m front-crawl performance, but chronotype was not consistently measured [10,19,20,21,22]. This leaves an important measurement gap: whether CSS itself varies with time of day in swimmers with a homogeneous chronotype when relevant pre-trial conditions are documented.

The rationale for examining this question is both methodological and task-specific. CSS is derived from the slope of the distance–time relationship between the 50 m and 400 m trials, which impose different energetic and technical demands. The 400 m effort and the sustainable-speed boundary represented by CSS have a greater oxidative contribution, whereas the approximately 26 s 50 m sprint depends more heavily on phosphagen and glycolytic energy supply, start and turn execution, stroke mechanics, and neuromuscular activation. Because time-of-day effects are less consistent for short-duration maximal exercise than for endurance performance [9,10], the two component trials may not change proportionally. A larger evening change in 400 m than in 50 m performance could therefore alter the calculated CSS even without a true training adaptation. Restricting the sample to N-type swimmers reduces variability associated with extreme circadian preference, while documenting dietary intake, well-being, and forehead skin temperature helps contextualise session comparability. These design features strengthen the interpretation of a potential time-of-day shift but do not establish a circadian or temperature-mediated mechanism because circadian phase and core or muscle temperature were not measured directly.

Accordingly, the aim of this study was to compare CSS between 09:00 h and 17:00 h in well-trained male swimmers restricted to the N-type chronotype, with 50 m and 400 m performance examined as the component outcomes from which CSS was derived. We hypothesised that CSS would be higher and 400 m time faster in the evening, whereas 50 m time would show a smaller and less consistent difference.

## 2. Methods

### 2.1. Experimental Design

A within-subject, repeated-measures, randomised counter-balanced design was used. Each swimmer completed one session at 09:00 h and one at 17:00 h on separate days, with 48 h between sessions. Session order was counter-balanced. During the 48 h interval, swimmers continued their habitual club training rather than following a standardised training load; this is acknowledged as a limitation. During the 24 h before each session, participants maintained habitual activity and diet and avoided caffeine, ergogenic supplements, alcohol, and vigorous non-training exercise. They were also asked to maintain their habitual sleep–wake schedule, although sleep timing and duration were not recorded objectively. Forehead skin temperature, resting heart rate, perceived recovery, and 24 h energy and carbohydrate intake were monitored as potential explanatory variables. Both sessions took place in the same indoor 25 m pool (depth 2 m; water temperature 26.5 °C), within the FINA-approved competition range of 25–28 °C. The Trakya University Ethics Committee approved the protocol (1 April 2024; protocol TUTF-GOBAEK 2024/137), and the study was conducted in accordance with the Declaration of Helsinki. Written informed consent was obtained from each participant and, for minors, from a parent or legal guardian.

### 2.2. Participants

An a priori sample-size calculation was performed in G*Power 3.1.9.4 (Heinrich Heine University, Düsseldorf, Germany) for a two-tailed paired-samples t test, with α = 0.05, power (1 − β) = 0.80, and an assumed Cohen’s dz = 0.80. The calculation indicated a minimum of 15 participants [23]. This large assumed effect was informed by previously reported morning-to-evening differences of approximately 1.2–3.5% in front-crawl performance [10,19,20]. We enrolled 16 participants to allow for attrition or missing data. Of 24 well-trained male swimmers screened, 16 met the pre-defined N-type criterion (tMEQ 42–58 AU) and were enrolled. Five moderate-evening-type and three moderate-morning-type swimmers were excluded; no swimmer was classified as definite-evening or definite-morning. Because the observed CSS effect (d_av = 0.24) was smaller than the planning assumption, the study was powered to detect a large paired effect but not to exclude smaller effects.

The retained N-type cohort comprised 16 male swimmers (age 17 ± 1 yr; height 1.78 ± 0.03 m; body mass 69.18 ± 4.56 kg) from one swimming club. Participants were classified as Tier 2–3 athletes (*n* = 4 Tier 2, trained/developmental; *n* = 12 Tier 3, highly trained/national level) according to McKay et al. [24]. All had at least 10 years of competitive swimming experience and at least 5 years of participation in regional- and national-level events. Their training included 6–8 in-water sessions per week (12–16 h·wk^−1^; approximately 45 km·wk^−1^) and 2–3 dry-land sessions per week (2–3 h·wk^−1^). Personal-best 400 m freestyle performances corresponded to 756 ± 28 FINA points.

### 2.3. Chronotype Assessment

Chronotype was assessed during the familiarisation visit using the validated Turkish version of the Horne–Östberg Morningness–Eveningness Questionnaire (tMEQ; test–retest reliability 0.84) [25]. To standardise administration, participants completed the questionnaire at 14:00 h ± 60 min, outside both experimental windows. The 19-item tMEQ yields a score from 16 to 86: 16–30, definite evening; 31–41, moderate evening; 42–58, neither/intermediate (N-type); 59–69, moderate morning; and 70–86, definite morning. Only swimmers classified as N-type were retained. The distribution is reported in Section 3.1 and Table 1.

### 2.4. Twenty-Four-Hour Dietary Intake

Pre-trial 24 h energy and macronutrient intake were recorded by direct visual observation rather than self-report, thereby reducing recall and social-desirability bias [26]. A trained researcher recorded every food and beverage consumed during the 24 h before each session, together with portion sizes estimated using household measures and a calibrated digital food scale (±1 g). The records were provided to a registered dietitian, independent of the testing team, who manually quantified energy intake (kcal·d^−1^), carbohydrate intake (g·d^−1^ and kcal·d^−1^), protein, and fat using a national food-composition database. The dietitian was blinded to session order and trial outcomes.

### 2.5. Measurements on the Test Day

Upon arrival, perceived recovery was assessed using the Hooper Index, a four-item 7-point Likert scale covering sleep quality, stress, fatigue, and muscle soreness; 1 represents the most favourable and 7 the least favourable response [27,28]. The total score was the sum of the four subscales. Heart rate was measured at rest and immediately after each swim using an optical sensor positioned in the swim cap (Polar OH1+, Polar Electro Oy, Kempele, Finland) in swim mode. Forehead skin temperature was measured in triplicate with a non-contact infrared thermometer (Jumper JPD-FR202, Shenzhen Jumper, Shenzhen, China). Resting forehead skin temperature and resting heart rate were measured once per session, on arrival and before the standardised warm-up; they were not measured separately immediately before the 50 m and 400 m trials. Borg CR-10 (0–10) RPE [29], which has been evaluated in competitive swimmers [30], was recorded immediately after each trial.

### 2.6. Critical Swimming Speed (CSS) Test

Each session began with approximately 10 min of land-based mobility and 25 min of in-water aerobic and pace work. During familiarisation, at least 48 h before the first experimental session, each swimmer completed one practice 50 m trial and one practice 400 m trial under the same warm-up and recovery conditions. Five minutes after the warm-up, participants performed a 50 m all-out front-crawl trial, followed by 30 min of passive seated recovery. Only water was permitted during recovery, and the volume consumed was recorded and replicated in the second session. Participants then completed a 400 m all-out front-crawl trial. All swims used a wall start and were timed electronically with a FINA-approved Daktronics touch-pad system (Daktronics Inc., Brookings, SD, USA) operated by a national-level coach. The system has a resolution of 0.01 s and avoids the error associated with handheld timing [31]. Trials were completed individually to minimise pacing and competitive effects.

CSS was calculated from the 50 m and 400 m distance–time data using the two-distance linear model applied to front-crawl swimming by Dekerle et al. [32]: CSS (m·s^−1^) = (D_2_ − D_1_)/(T_2_ − T_1_), where D_1_ = 50 m, D_2_ = 400 m, and T_1_ and T_2_ are the corresponding times [32,33]. The two-distance protocol was selected to limit cumulative fatigue, athlete burden, and pool time in a repeated-measures design. The 50 m and 400 m trials also bracketed the sprint and aerobic performance ranges relevant to the study hypothesis; adding 100 m and 200 m trials would not have widened this distance span. Nevertheless, a two-distance CSS estimate is sensitive to the selected anchors, and future studies should consider three-distance and split-level analyses.

### 2.7. Statistical Analysis

Analyses were performed in JASP (v0.19.2; JASP Team, University of Amsterdam, Amsterdam, The Netherlands). Normality of the paired morning–evening differences was assessed with the Shapiro–Wilk test and was satisfied for every dependent variable (all *p* > 0.05). Two-tailed paired-samples t tests were therefore used. Statistical significance was set a priori at *p* < 0.05, and data are presented as mean ± standard deviation (SD). Mean differences (Δ) were calculated as evening minus morning; negative differences for time outcomes therefore indicate faster evening performance. Reported Cohen’s d_av values were calculated as (M_evening − M_morning)/√[(SD_morning^2^ + SD_evening^2^)/2], with 0.20, 0.50, and 0.80 interpreted as small, medium, and large, respectively [34]. CSS was the sole pre-specified primary outcome. The 50 m and 400 m times were secondary outcomes and also constituted the CSS calculation; physiological, perceptual, well-being, and dietary measures were exploratory. No multiplicity correction was applied to secondary or exploratory outcomes, which should therefore be interpreted as hypothesis-generating. Table 2 and Table 3 report Δ, effect size, *p* value, and the available 95% confidence intervals. Session order was counter-balanced, but a session × order interaction was not formally modelled.

## 3. Results

### 3.1. Chronotype Distribution of the Sample

All 16 enrolled swimmers met the pre-defined N-type inclusion criterion. The mean tMEQ score was 49.68 ± 4.86 AU (range 43–57 AU), and every score fell within the neither/intermediate band (42–58 AU). No swimmer in the analysis sample was classified as morning-type or evening-type (Table 1).

### 3.2. Pre-Trial Well-Being and 24 h Dietary Intake

Neither the Hooper Index total nor any of its four subscales differed significantly between morning and evening (all *p* > 0.05; Table 2). Fatigue showed the largest descriptive difference (Δ = −0.47 AU; d_av = −0.42), but this comparison was non-significant (*p* = 0.072). Energy intake (3496.3 ± 398.8 vs. 3529.4 ± 380.2 kcal·d^−1^; *p* = 0.761) and carbohydrate intake (2097.8 ± 239.3 vs. 2117.8 ± 228.1 kcal·d^−1^; *p* = 0.812) were also non-significant.

### 3.3. Swimming Performance and Physiological Responses

**Figure 1:**
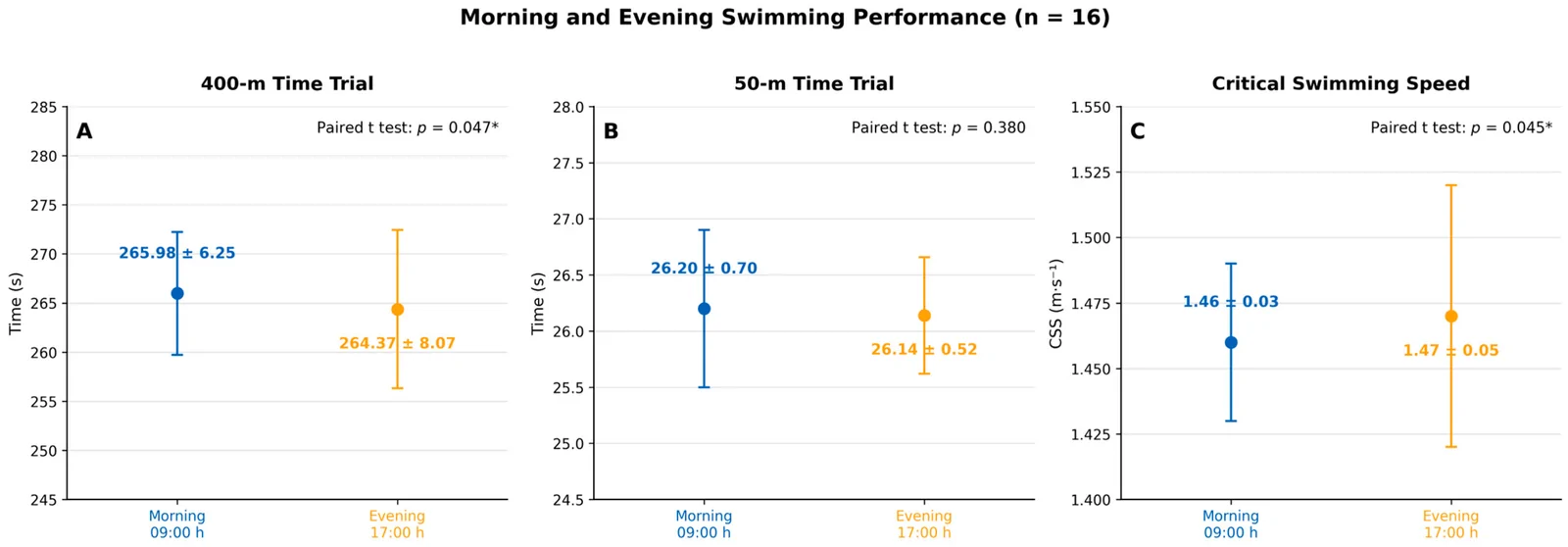
Group performance at 09:00 h (morning; blue) and 17:00 h (evening; orange) in well-trained N-type male swimmers (*n* = 16). (**A**) 400-m time trial, (**B**) 50-m time trial, and (**C**) critical swimming speed (CSS). Points show means; error bars show SD. Paired-samples *t*-tests showed significant differences between morning and evening for 400-m time (*p* = 0.047) and CSS (*p* = 0.045), but not for 50-m time (*p* = 0.380). *: *p* < 0.05.

The 400 m time was significantly faster in the evening than in the morning (morning, 265.98 ± 6.25; evening, 264.37 ± 8.07 s; Δ = −1.61 s [−0.61%]; *p* = 0.047; Figure 1A). The 50 m time did not differ between sessions (morning, 26.20 ± 0.70; evening, 26.14 ± 0.52 s; Δ = −0.06 s [−0.23%]; *p* = 0.380; Figure 1B). With *n* = 16 and an observed |d_av| of approximately 0.10, the non-significant 50 m result does not establish equivalence or exclude a small effect. CSS, the pre-specified primary outcome, was significantly higher in the evening (morning, 1.46 ± 0.03; evening, 1.47 ± 0.05 m·s^−1^; Δ = +0.01 m·s^−1^ [+0.68%]; *p* = 0.045; d_av = 0.24; Figure 1C; Table 3).

Forehead skin temperature was higher in the evening than in the morning (morning, 36.2 ± 0.2; evening, 36.8 ± 0.3 °C; *p* < 0.001; d_av = 2.35), as was resting heart rate (morning, 65 ± 6; evening, 71 ± 5 bpm; *p* < 0.001; d_av = 1.10). Post-50 m heart rate (morning, 182.6 ± 7.3; evening, 178.5 ± 9.4 bpm; *p* = 0.068) and post-400 m heart rate (morning, 187.1 ± 3.9; evening, 189.4 ± 10.3 bpm; *p* = 0.450) did not differ significantly. Borg CR-10 RPE was also non-significant after the 50 m trial (morning, 6.87 ± 1.62; evening, 6.75 ± 1.44 AU; *p* = 0.844) and the 400 m trial (morning, 7.50 ± 1.21; evening, 7.56 ± 1.50 AU; *p* = 0.900; Table 3).

## 4. Discussion

The present study yielded three principal findings in 16 well-trained, neither/intermediate-type (N-type) adolescent male front-crawl swimmers. First, the pre-specified primary outcome, critical swimming speed (CSS), was modestly higher in the evening (1.46 ± 0.03 vs. 1.47 ± 0.05 m·s^−1^; Δ = +0.68%; d_av = 0.24; *p* = 0.045), and the secondary 400 m time trial was faster (265.98 ± 6.25 vs. 264.37 ± 8.07 s; Δ = −0.61%; d_av = −0.22; *p* = 0.047). Second, the all-out 50 m sprint did not differ (26.20 ± 0.70 vs. 26.14 ± 0.52 s; d_av = −0.10; *p* = 0.380). With *n* = 16, this non-significant result neither establishes equivalence nor excludes a small effect; generalisability is further limited by the exclusively adolescent N-type male cohort. Third, the performance differences accompanied higher evening forehead skin temperature (+0.6 °C) and resting heart rate (+6 bpm), whereas perceived well-being and dietitian-verified 24 h energy and carbohydrate intake were unchanged. The non-significant fatigue trend (d_av = −0.42; *p* = 0.072) remains a possible residual confounder. Thus, the pattern supports a small time-of-day difference in CSS and 400 m performance, but not sensitivity to circadian phase or a causal mechanism. The 0.68% CSS difference lies below the approximately 1.2–3.5% morning-to-evening variation reported for 25–200 m front-crawl performance [10,19,20,21,22] and accords with smaller variation in longer aerobic tasks after chronotype and prior activity are considered [35,36].

Despite its small magnitude, a systematic difference of approximately 0.7% can complicate repeated testing. It is comparable to smallest-worthwhile-change estimates of approximately 0.3–0.5% for middle-distance freestyle [37], although this indicates measurement sensitivity rather than proven competitive importance, particularly given the wide confidence interval and modest effect. Pre- and post-intervention tests should therefore be scheduled at the same time of day [38]. Chronotype may partly explain the larger amplitudes in earlier studies, where it was often unmeasured or unbalanced [15,36,39]. Restricting the present sample to N-type swimmers reduced, but did not remove, this heterogeneity; the approximately 0.7% difference is therefore a cohort-specific reference for similarly trained N-type adolescent males, not an estimate for all swimmers [39]. Habitual training at a specific time may further modify the response [36,40]. Anderson et al. [7] reported that evening-type collegiate swimmers were approximately 6% slower in the morning and that morning types were impaired in the evening. The present data extend this work by demonstrating a smaller evening advantage in N-type swimmers. Because that study did not characterise training volume, our Tier 2–3 cohort (approximately 45 km·wk^−1^; regional/national competition) provides a useful reference for highly trained adolescents. Chronotype screening, dietary assessment, forehead skin temperature, and resting heart rate reduce several alternative explanations but cannot show that the shift was circadian rather than substrate-driven. The findings are nevertheless consistent with a proposed graded relationship between performance amplitude and chronotype–testing-time mismatch, which requires direct testing in mixed-chronotype samples [15,36,39].

The unchanged 50 m sprint but faster evening 400 m performance and higher CSS are compatible with the trials’ distinct demands. The approximately 26 s 50 m sprint depends on rapid phosphocreatine breakdown, capacity-limited glycolytic flux, neuromuscular activation, stroke mechanics, and start and turn execution [9,41,42]. These determinants show smaller and less consistent time-of-day effects [9,41,42]. The present protocol did not address jet-lag-related circadian disruption [43]. The 400 m trial and CSS have a greater oxidative contribution and relate conceptually to maximal-metabolic-steady-state and critical-power frameworks [3,44]. Time of day and chronotype can also influence hormonal responses to high-intensity exercise [45]. Cross-disciplinary evidence indicates a more consistent afternoon advantage for maximum endurance than maximum strength [41], with evening benefits for some jump and agility outcomes but not all explosive sprints [46]. Forehead skin temperature and resting heart rate were measured once on arrival before warm-up, so the same higher evening session-level value preceded both trials. The divergent outcomes may reflect different task sensitivities, but cannot show that temperature affected one trial more than the other. The 0.6 °C surface-temperature elevation is directionally consistent with the late-afternoon core-temperature peak [35,47,48], yet forehead skin temperature is not a direct measure of core or muscle temperature. General models propose that warming may enhance oxidative-enzyme kinetics (Q_10_ approximately 2.0), cross-bridge cycling, fibre-conduction velocity, and oxygen unloading through the Bohr effect [7,11,13,35,47,48]. Temperature, hormonal rhythms, and chronotype may therefore interact in aerobic performance [49,50], and experimental warming of morning muscle towards evening levels has attenuated diurnal variation in force production [47,51]. These observations make a temperature-related explanation plausible, not proven. Likewise, the 6 bpm higher resting heart rate, with unchanged post-trial heart rate and RPE, is compatible with altered autonomic tone rather than acute sympathetic overshoot [35,45,52].

Another strength was concurrent assessment of chronotype, pre-trial nutrition, and body-surface temperature. Energy intake (Δ = +33.1 kcal·d^−1^; d_av = 0.08; *p* = 0.761) and carbohydrate intake (Δ = +20.0 kcal·d^−1^; d_av = 0.09; *p* = 0.812) were nearly identical, based on direct observation and blinded dietitian analysis [26]. This reduces the likelihood of a large measured nutritional imbalance, without excluding all differences in substrate availability. Borg CR-10 RPE was similar after the 50 m (6.87 ± 1.62 vs. 6.75 ± 1.44 AU; *p* = 0.844) and 400 m trials (7.50 ± 1.21 vs. 7.56 ± 1.50 AU; *p* = 0.900), and post-trial heart rate was also non-significant [29,30]. Ratings near 7 (‘very hard’) and heart rates of approximately 178–189 bpm are compatible with strong effort, but do not independently verify maximality. Faster 400 m performance at similar RPE and post-trial heart rate is consistent with a higher tolerable external work rate at a comparable measured internal load. This accords with the critical-power framework, in which a higher critical-speed boundary permits faster sustainable swimming [44]. Evening performance or recovery advantages at matched exertion have also been reported in soccer [53] and tennis [54], whereas anaerobic responses remain task-dependent [55]. These comparisons support an aerobically weighted interpretation, but do not identify the mechanism.

Overall, a small evening increase in CSS accompanied a faster secondary 400 m time in this N-type male cohort. The wide confidence intervals, small sample, and uncorrected secondary and exploratory analyses require caution. Similar measured diet, well-being, post-trial heart rate, and RPE do not prove identical sessions; sleep timing was not objectively recorded, training load was not standardised, and maximality was not independently verified by blood lactate or oxygen uptake. Nor did the study assess travel-related circadian disruption, sleep loss, or internal desynchronisation, so the findings should not be extrapolated to jet lag [43]. They primarily support standardised test scheduling, not universal evening training or a numerical correction factor.

### 4.1. Practical Applications

Four provisional implications follow. First, the time of day should be matched across repeated CSS tests and, where relevant, with the targeted training stimulus [38,56,57]. Second, when a fixed window is needed, approximately 17:00 h may be pragmatic for cohorts resembling this sample, but is not universally optimal. Third, chronotype should be screened with the tMEQ and the habitual training time should be documented [25,58,59]. Fourth, the approximately 0.7% difference should be treated only as a sensitivity check in comparable N-type swimmers, not as a universal downward correction to CSS-derived pace in double-day training.

### 4.2. Strengths and Limitations

The study’s strengths include a within-subject counter-balanced design with a 48 h interval, a homogeneous N-type cohort, direct-observation dietary assessment, a standardised warm-up, electronic timing, and concurrent measurement of well-being, forehead skin temperature, and heart rate. Together, these features address several of the principal sources of variability identified in the time-of-day literature [35,36,38,39]. The following limitations should nonetheless be acknowledged.

First, the cohort was small, exclusively male, drawn from one club, restricted to the N-type chronotype, and not formally staged for maturation. The observed amplitude should therefore not be generalised to female swimmers or to athletes with morning or evening chronotypes [7,36,39,60].

Second, several physiological measures were indirect or incomplete. Forehead skin temperature is a surrogate that can differ from core temperature by approximately 0.3–0.5 °C, an error magnitude similar to the observed 0.6 °C session difference [61]. The temperature-based mechanisms discussed above are therefore inferred rather than directly established. Core and muscle temperature, blood lactate, salivary cortisol and testosterone, dim-light melatonin onset, and objective sleep measures were not obtained; sleep was characterised only by the subjective Hooper subscale.

Third, the two-distance 50 m/400 m CSS model produces a distance-sensitive point estimate, does not provide the same modelling redundancy as a three-distance protocol, and did not include split-level analysis of 400 m pacing [1,33,44].

Fourth, several operational and statistical controls were incomplete. The 48 h interval allowed uncontrolled variation in lifestyle; training load was not standardised; the supervising coach was not blinded to session time; within-cohort CSS reliability was not re-estimated [1]; and a session × order interaction was not modelled. The study was powered for a large paired effect, whereas the observed CSS effect was small. Consequently, the non-significant 50 m result indicates only that no effect was detected here; it does not establish equivalence. Finally, CSS was the sole pre-specified primary outcome. The nominally significant 400 m result was secondary, and the physiological, perceptual, well-being, and dietary comparisons were exploratory and uncorrected for multiplicity. These findings should therefore be regarded as hypothesis-generating pending independent confirmation.

### 4.3. Future Research Directions

Future studies should include larger, sex-balanced samples and morning-, evening-, and N-type swimmers tested at both times to estimate the chronotype × time-of-day interaction [36,39]. Intramuscular or gastrointestinal thermometry, blood-lactate kinetics, salivary cortisol and testosterone, dim-light melatonin onset, and actigraphy would help distinguish temperature, autonomic, and hormonal pathways [11,35,45,52]. Longitudinal comparisons of identical training at 09:00 and 17:00 h should test whether time-specific training changes CSS amplitude [40,62]. Research should also examine trans-meridian disruption and light-, melatonin-, or exercise-based countermeasures [43]. Replications should add a three-distance protocol with D′ estimation, split-level pacing, repeated-sprint outcomes [63], blinded assessment, an internal reliability substudy, and order-effect modelling. Chronotype- and training-time-anchored decision tools may ultimately help coaches, but correction factors require independent validation.

## 5. Conclusions

In well-trained adolescent male swimmers with a homogeneous N-type chronotype, CSS was modestly higher, and 400 m performance was faster at 17:00 h than at 09:00 h, whereas 50 m performance did not differ. Forehead skin temperature and resting heart rate were also higher in the evening, but these concurrent changes do not establish a physiological mechanism. The small sample, secondary status of the 400 m outcome, and unadjusted exploratory analyses limit the strength and generalisability of the conclusions. Repeated CSS assessments should be performed at a consistent time of day; the present data do not justify a universal numerical correction. Larger, sex-balanced, and chronotype-diverse studies using direct physiological measurements are required.

## Figures and Tables

**Table 1:** Distribution of Horne–Östberg chronotype categories in the analysis sample (*n* = 16).

Chronotype Category (tMEQ Score Range)	*n*	% of Sample	Mean tMEQ Score ± SD	Range (min–max)
Definite evening type (16–30)	0	0.0	–	–
Moderate evening type (31–41)	0	0.0	–	–
Neither/intermediate type (42–58)	16	100.0	49.68 ± 4.86	43–57
Moderate morning type (59–69)	0	0.0	–	–
Definite morning type (70–86)	0	0.0	–	–
Sample total	16	100.0	49.68 ± 4.86	43–57

tMEQ, Turkish version of the Horne–Östberg Morningness–Eveningness Questionnaire; SD, standard deviation. Classification cut-offs follow Pündük et al. [25].

**Table 2:** Diurnal comparison of perceived well-being and 24 h dietary intake (*n* = 16).

Variable	Morning @ 09:00 (mean ± SD)	Evening @ 17:00 (mean ± SD)	Cohen’s d_av	*p*-Value	Δ
Perceived sleep quality (AU)	2.80 ± 1.26	2.73 ± 1.16	−0.05	0.783	−0.07
Stress (AU)	2.60 ± 0.74	2.47 ± 0.83	−0.16	0.571	−0.13
Fatigue (AU)	3.87 ± 1.13	3.40 ± 1.06	−0.42	0.072	−0.47
Muscle soreness (AU)	3.80 ± 1.21	3.73 ± 1.16	−0.06	0.796	−0.07
Hooper Index total (AU)	13.00 ± 3.38	12.33 ± 3.06	−0.21	0.154	−0.67
Total energy intake (kcal·d^−1^)	3496.3 ± 398.8	3529.4 ± 380.2	0.08	0.761	+33.1
Carbohydrate intake (kcal·d^−1^)	2097.8 ± 239.3	2117.8 ± 228.1	0.09	0.812	+20.0

AU, arbitrary units; SD, standard deviation. Δ and Cohen’s d_av are presented as evening minus morning. Recalculation from the displayed rounded statistics may differ by approximately 0.01. Effect-size thresholds were 0.20 (small), 0.50 (medium), and 0.80 (large). Paired-mean-difference 95% confidence intervals (evening − morning): sleep quality, −0.60 to 0.46 AU; stress, −0.61 to 0.35 AU; fatigue, −0.99 to 0.05 AU; muscle soreness, −0.64 to 0.50 AU; Hooper Index total, −1.62 to 0.28 AU; energy intake, −195 to 261 kcal·d^−1^; and carbohydrate intake, −156 to 196 kcal·d^−1^.

**Table 3:** Diurnal comparison of swimming performance, forehead skin temperature, heart rate, and rating of perceived exertion (*n* = 16).

Variable	Morning @ 09:00 (mean ± SD)	Evening @ 17:00 (mean ± SD)	Cohen’s d_av	*p*-Value	Δ
50 m time (s)	26.20 ± 0.70	26.14 ± 0.52	−0.10	0.380	−0.06
400 m time (s)	265.98 ± 6.25	264.37 ± 8.07	−0.22	0.047 *	−1.61
CSS (m·s^−1^)	1.46 ± 0.03	1.47 ± 0.05	0.24	0.045 *	+0.01
Forehead temperature (°C)	36.2 ± 0.2	36.8 ± 0.3	2.35	<0.001 *	+0.6
Resting HR (bpm)	65 ± 6	71 ± 5	1.10	<0.001 *	+6
HR post-50 m (bpm)	182.6 ± 7.3	178.5 ± 9.4	−0.49	0.068	−4.1
HR post-400 m (bpm)	187.1 ± 3.9	189.4 ± 10.3	0.30	0.450	+2.3
RPE post-50 m (Borg CR-10 AU)	6.87 ± 1.62	6.75 ± 1.44	−0.08	0.844	−0.12
RPE post-400 m (Borg CR-10 AU)	7.50 ± 1.21	7.56 ± 1.50	0.04	0.900	+0.06

CSS, critical swimming speed; HR, heart rate; RPE, rating of perceived exertion; AU, arbitrary units; SD, standard deviation. * *p* < 0.05. Δ and Cohen’s d_av are signed as evening minus morning; negative time differences denote faster evening performance. Recalculation from the displayed rounded statistics may differ by approximately 0.01. Paired-mean-difference 95% confidence intervals (evening − morning): 50 m time, −0.20 to 0.08 s; 400 m time, −3.20 to −0.02 s; CSS, 0.000 to 0.019 m·s^−1^ (the lower limit was positive before rounding); HR post-50 m, −8.5 to 0.3 bpm; HR post-400 m, −4.0 to 8.6 bpm; RPE post-50 m, −1.40 to 1.16 AU; and RPE post-400 m, −0.94 to 1.06 AU. Session-mean 95% confidence intervals were 36.1 to 36.3 °C (morning) and 36.6 to 37.0 °C (evening) for forehead skin temperature, and 62 to 68 bpm (morning) and 68 to 74 bpm (evening) for resting heart rate. Paired-samples t-test *p*-values are reported in the table.

## Data Availability

The data supporting the findings of this study are available from the corresponding author upon reasonable request.
